# Analysis of metabolites in serum and villous tissue of missed abortion patients based on targeted metabolomics

**DOI:** 10.3389/fendo.2026.1782638

**Published:** 2026-06-18

**Authors:** Huan Zhao, Wei Liu, Doudou Zhao, Mi Zhang, Muxi Yang, Li Shan

**Affiliations:** Department of Gynecology, Northwest Women’s and Children’s Hospital, Xi’an, Shaanxi, China

**Keywords:** acylcarnitine, amino acid, metabolomics, missed abortion, serum, villous tissue

## Abstract

**Objective:**

In recent years, advancements in metabolomics have highlighted that changes in metabolic states within the body might also be closely related to missed abortion. However, the specific impacts of metabolic abnormalities on missed abortion remain unclear. This study screened differential metabolites in serum and villous tissues from patients with missed abortion, analyzed possible mechanisms linking metabolic disturbances to disease occurrence, and explored their clinical research value in early pregnancy abnormality research.

**Methods:**

Focusing on the effect of abnormal levels of metabolites on missed abortion, a prospective case–control study was conducted including 78 women with missed abortion and 75 women with normal early pregnancy undergoing elective termination. Targeted metabolomics profiling of serum and villous tissues was performed using mass spectrometry. Clinical characteristics and metabolite levels were compared between groups. Firth’s logistic regression was applied to assess independent associations. ROC curve analysis was used to evaluate the discriminatory potential of key metabolites.

**Results:**

Results revealed metabolic differences in both serum and villous tissues between the missed abortion and control groups. Serum was characterized by upregulated amino acid metabolism, with elevated levels of multiple amino acids including ORN and TYR, which were associated with the risk of missed abortion. Villous tissues exhibited core disturbances in acylcarnitine metabolism, with C10:1 and C18 significantly upregulated in the missed abortion group. Notably, no highly consistent differential metabolites were identified between serum and villous tissues.

**Conclusion:**

This study demonstrates distinct metabolic signatures in missed abortion, with amino acid upregulation in serum and acylcarnitine disorder in villous tissues. Only CIT was consistently upregulated in both matrices, possibly due to limited assay coverage. Combined panels of the top eight FDR-ranked metabolites achieved high AUCs in serum and villous tissue, offering good auxiliary discrimination for missed abortion. These findings provide new insights into its metabolic pathogenesis.

## 1 Introduction

Missed abortion, defined as the cessation of embryo development or death in the uterine cavity during early pregnancy, with the embryo remaining in the uterus without being naturally expelled, is one of the most common adverse pregnancy outcomes. In recent years, the incidence of missed abortion has been on the rise, accounting for about 15% of natural miscarriages in clinical pregnancies (1).Missed abortion is primarily diagnosed by ultrasonography. Typical ultrasonic features involve irregular gestational sac shape, absent fetal heart activity, and arrested embryonic development. Serial monitoring of serum HCG can also be used for auxiliary diagnosis, with affected patients presenting with slowly increasing or progressively decreasing HCG concentrations.

It is generally believed that missed abortion has complex and diverse causes and may result from a combination of multiple factors. Studies have found that fetal chromosomal abnormalities, including numerical and structural aberrations, are the main causative factors for missed abortion. Among these, chromosomal aneuploidy is a significant cause of missed abortions (2). Moreover, the abnormal expression of maternal autoimmune antibodies such as anti-sperm antibodies and anti-cardiolipin antibodies is often closely related to miscarriage. Research has also found that an abnormal increase in Ureaplasma urealyticum and Chlamydia trachomatis in the female reproductive tract can also be a significant cause of early miscarriages. In addition, factors such as age, bad life habits and other external influences can lead to missed abortion in pregnant women (3). However, despite being commonly associated with factors such as immune, genetic, infectious, environmental, endocrine, and socio-psychological influences, the cause remains unknown in 50% of cases of missed miscarriages. Therefore, conducting thorough and comprehensive research into the causes of missed abortion and finding more accurate preventive and diagnostic indicators is an urgent task.

Metabolomics, which profiles small-molecule metabolites reflecting real-time physiological and pathological states, has emerged as a powerful tool in obstetric research, particularly for gestational diabetes, assisted reproductive technologies, polycystic ovary syndrome, endometriosis, ovarian cancer biomarkers, and preeclampsia (4–12). Among studies related to abortion and metabolism, there are reports indicating differences in plasma metabolites between patients with ectopic pregnancy and those with intrauterine pregnancy abortions. Studies have also shown differences in the metabolites found in the serum and plasma of pregnant women with missed abortions compared to those undergoing normal induced abortions (12–14). However, existing studies are limited to single matrices (serum/plasma) and lack paired serum–villous tissue validation, leaving unclear whether metabolic disturbances are systemic or localized at the maternal–fetal interface. To address these limitations, this study performed targeted metabolomics analysis of paired serum and villous tissue samples from women with missed abortion and normal early pregnancy. We aimed to explore systemic and local metabolic perturbations, identify key differential metabolites, and assess the discriminatory capacity of metabolite combinations. The findings may provide insights into the metabolic dysregulation underlying missed abortion and serve as a reference for further mechanistic studies.

## 2 Materials and methods

### 2.1 Research participants

This study involved screening patients with missed abortions who met the inclusion criteria and were treated at the Northwest Women’s and Children’s Hospital from March 2024 to December 2024. A total of 78 cases were included in the experimental group, while 75 cases of normal early pregnancy requiring termination were included in the control group.

### 2.2 Diagnostic criteria

Patients with early missed abortion who met the diagnostic criteria in the 2020 Expert Consensus on the Treatment of Early Missed Abortion were selected. The inclusion criteria were as follows (1): Ultrasound showing a crown-rump length (CRL) ≥ 7 mm without fetal heartbeat (2); ≥ 25 mm average diameter of the gestational sac in the uterine cavity without an embryo (3); No yolk sac observed in the uterine cavity during pregnancy, and no embryo or fetal heartbeat detected even after 2 weeks (4); A yolk sac visible in the uterine cavity during pregnancy, but no fetal heartbeat observed even after 11 days.

### 2.3 Mass spectrometry analysis

The tandem mass spectrometry system used for sample detection was the LC/MS/MS System (triple quadrupole mass spectrometer) from AB Sciex (model: API3200MD). The employed reagent kits included the Succinylacetone and Non-derivatized Multiple Amino Acids and Carnitine Detection Kit from Guangzhou Fenghua Biotechnology, among others.

### 2.4 Statistical methods

Statistical analyses were performed using SPSS 26.0 and R 4.2.1. Continuous variables are expressed as mean ± SD (normal distribution) or median (IQR) (non-normal distribution); categorical variables as n (%).Baseline characteristics between groups were compared using independent samples t-test or Mann-Whitney U test for continuous data, and chi-squared or Fisher’s exact test for categorical data, as appropriate.Differential metabolite levels were analyzed via Mann-Whitney U test, with false discovery rate (FDR) correction applied for multiple comparisons; FDR-adjusted P < 0.05 was considered significant.Firth penalized logistic regression was used to identify independent risk factors for missed abortion to address complete data separation issues. Receiver operating characteristic (ROC) curves were constructed to assess the diagnostic performance of individual metabolites and combined models, with the area under the curve (AUC) and 95% confidence intervals (CI) calculated. All tests were two-tailed, and P < 0.05 was considered statistically significant.

### 2.5 Inclusion and exclusion criteria

Inclusion Criteria: a) Patients with missed abortion at ≤ 12 weeks of gestation; all patients received medical abortion with mifepristone combined with misoprostol and achieved villus expulsion. Those who failed to expel villi medically underwent artificial abortion to obtain villus samples. b) Diagnosis consistent with the relevant diagnostic criteria specified in the 2020 Expert Consensus on the Treatment of Early Missed Abortion. c) All enrolled patients met the safety criteria for medication use, with no drug allergy or contraindications, and normal liver and renal function. d) Age ranged from 20 to 35 years old. e) Patients had normal mental status and intact verbal communication and comprehension ability. f) All participants were fully informed of the research content, volunteered to participate, and signed the informed consent form.

Exclusion Criteria: a) Patients with allergy or contraindications to prostaglandin drugs. b) Patients with a history of allergy or contraindications to mifepristone, such as those with endocrine diseases including adrenal diseases and diabetes. c) Patients with cardiac, hepatic, renal diseases or adrenal insufficiency. d) Individuals with a history of hematological diseases or hereditary porphyria. e) Anemic patients with hemoglobin concentration < 75 g/L indicated by routine blood examination. f) Patients diagnosed with or suspected of ectopic pregnancy, intrauterine device pregnancy, incomplete abortion, inevitable abortion, dilated cervical os, or other special abnormal pregnancy conditions. g) Cases lost to follow-up, with incomplete clinical data, or those with unevaluable therapeutic efficacy.

## 3 Results

### 3.1 Demographic data

A total of 153 subjects were included in this study, including 78 cases in the missed abortion group and 75 cases in the control group. This study included the general baseline data of patients in the missed abortion group and the control group: age, BMI, height, weight, average diameter of the gestational sac, and days of amenorrhea. Compared with women in the control group, those with the missed abortion group had a higher proportion of women aged < 35 years (98.72% vs. 80.00%), a higher BMI (22.43 kg/m² vs. 21.55 kg/m²), a larger mean gestational sac diameter (24.69 mm vs. 18.49 mm), and a longer duration of amenorrhea (67.45 days vs. 46.09 days) (**Table 1**).

**Table 1:** Patient demographics and baseline characteristics.

	Group			
Characteristic	Missed abortion, N = 78^1^	Control group, N = 75^1^	Statistic	P-value
age	31.05 ± 3.25	30.72 ± 5.47	0.453	0.651^2^
ageg			14.306	<0.001^3^
<35	77 (98.72%)	60 (80.00%)		
≥35	1 (1.28%)	15 (20.00%)		
height	161.67 ± 4.88	162.27 ± 4.15	-0.820	0.414^2^
weight	58.49 ± 6.29	56.65 ± 5.67	1.895	0.060^2^
BMI	22.43 ± 2.54	21.55 ± 2.41	2.190	0.030^2^
BMIg				0.108^4^
Underweight	3 (3.85%)	6 (8.00%)		
Normal	50 (64.10%)	55 (73.33%)		
Overweight and Obese	25 (32.05%)	14 (18.67%)		
Npregnanciesg			0.019	0.892^3^
1	31 (39.74%)	29 (38.67%)		
≥2	47 (60.26%)	46 (61.33%)		
Ndeliveriesg			3.433	0.064^3^
0	46 (58.97%)	33 (44.00%)		
≥1	32 (41.03%)	42 (56.00%)		
abortionsg			0.037	0.846^3^
0	54 (69.23%)	53 (70.67%)		
≥1	24 (30.77%)	22 (29.33%)		
Dgestationalsac	24.69 ± 10.48	18.49 ± 7.65	4.188	<0.001^2^
Dmenstrualcessation	67.45 ± 8.09	46.09 ± 7.51	16.924	<0.001^2^

^1^ Mean ± SD; n (%).

^2^ Welch Two Sample t-test.

^3^ Pearson’s Chi-squared test.

^4^ Fisher’s exact test.

### 3.2 Metabolomics analysis of serum and villous tissue obtained from the missed abortion and control groups

Targeted metabolomics was performed to quantify 11 amino acids and 31 acylcarnitines in the serum and villous tissues from missed abortion and control group subjects.

#### 3.2.1 Serum

**Figure 1:**
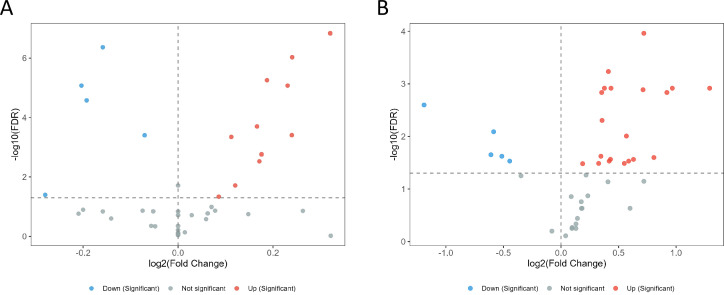
**(A)** Volcano plot for differential metabolites in serum. **(B)** Volcano plot for differential metabolites in villous tissues.The abscissa represents log_2_(fold change), and the ordinate represents -log_10_(FDR). Red dots indicate metabolites significantly elevated in the missed abortion group (FDR < 0.05 and FC > 1), blue dots represent significantly decreased metabolites (FDR < 0.05 and FC < 1), and gray dots denote metabolites with no statistically significant difference. The horizontal dashed line indicates the significance threshold of FDR = 0.05, and the vertical middle line represents the change threshold of log_2_FC = 0.

Compared with control group, missed abortion patients showed higher levels of ALA, ARG, CIT, LEU+ILE+ProOH, ORN, PHE, PRO, TYR, VAL, C3, C5 and C18, and lower levels of C4DC/C5OH, C6, C12, C14, C14OH and C16OH (P < 0.05). After false discovery rate (FDR) correction, significant differential expression was observed for 17 metabolites between the missed abortion group and the control group (FDR < 0.05), whereas the other 25 metabolites displayed no statistically significant intergroup differences (FDR ≥ 0.05). Among these differentially expressed metabolites, amino acids including ORN, TYR, LEU+ILE+ProOH, ALA, VAL, PRO, CIT, ARG, and PHE exhibited elevated levels in the missed abortion group, with ORN, TYR, and LEU+ILE+ProOH exhibiting the most pronounced alterations. Additionally, certain carnitines C6, C16OH, C14, and C12 showed reduced levels in the missed abortion group, while other carnitine species, namely C3, C4DC/C5OH, C5, and C14OH, also presented significant intergroup expression differences (**Table 2**; **Figure 1A**).

**Table 2:** Differential metabolites in the serum.

Characteristic	Missed abortion, N = 78^1^	Control group, N = 75^1^	P-value	FDR
ALA	179.890 ± 33.189	153.235 ± 28.413	<0.001^2^	<0.001
ARG	39.061 (34.168, 42.332)	36.804 (32.713, 39.649)	0.018^3^	0.046
CIT	8.978 (7.783, 10.027)	7.947 (7.032, 8.725)	<0.001^3^	0.002
GLY	132.359 (112.054, 157.136)	145.952 (112.382, 176.062)	0.185^3^	0.250
LEU+ILE+ProOH	88.957 (80.521, 96.941)	78.115 (68.601, 84.701)	<0.001^3^	<0.001
MET	10.572 (9.842, 11.595)	10.364 (9.526, 11.022)	0.137^3^	0.192
ORN	42.327 (36.715, 51.044)	33.889 (30.132, 39.060)	<0.001^3^	<0.001
PHE	38.064 (34.558, 40.909)	33.802 (31.325, 39.753)	<0.001^3^	0.003
PRO	59.814 (49.538, 70.519)	50.652 (45.383, 55.808)	<0.001^3^	<0.001
TYR	28.554 (24.547, 31.899)	24.167 (21.429, 26.846)	<0.001^3^	<0.001
VAL	102.652 ± 16.890	90.473 ± 15.459	<0.001^2^	<0.001
C0	12.823 (11.464, 14.144)	12.280 (10.764, 13.612)	0.104^3^	0.169
C2	2.986 (2.362, 3.943)	3.087 (2.498, 3.989)	0.380^3^	0.456
C3	0.281 (0.262, 0.304)	0.260 (0.241, 0.283)	<0.001^3^	<0.001
C3DC/C4OH	0.027 (0.023, 0.030)	0.028 (0.025, 0.034)	0.078^3^	0.143
C4	0.057 (0.050, 0.069)	0.057 (0.047, 0.065)	0.359^3^	0.444
C4DC/C5OH	0.040 (0.037, 0.042)	0.042 (0.040, 0.044)	<0.001^3^	<0.001
C5	0.050 (0.045, 0.055)	0.046 (0.043, 0.052)	0.007^3^	0.019
C5.1	0.006 (0.005, 0.007)	0.005 (0.005, 0.006)	0.072^3^	0.138
C5DC/C6OH	0.138 (0.124, 0.151)	0.138 (0.128, 0.147)	0.704^3^	0.771
C6	0.129 (0.120, 0.137)	0.144 (0.136, 0.150)	<0.001^3^	<0.001
C6DC	0.202 (0.189, 0.211)	0.200 (0.188, 0.214)	0.644^3^	0.731
C8	0.042 (0.030, 0.066)	0.048 (0.038, 0.070)	0.111^3^	0.172
C8.1	0.063 (0.048, 0.085)	0.060 (0.044, 0.081)	0.200^3^	0.263
C10	0.047 (0.034, 0.068)	0.054 (0.044, 0.079)	0.057^3^	0.126
C10.1	0.075 (0.062, 0.084)	0.079 (0.066, 0.096)	0.066^3^	0.136
C10.2	0.008 (0.006, 0.009)	0.008 (0.007, 0.010)	0.082^3^	0.144
C12	0.014 (0.009, 0.019)	0.017 (0.012, 0.023)	0.015^3^	0.040
C12.1	0.022 (0.017, 0.027)	0.024 (0.020, 0.027)	0.086^3^	0.144
C14	0.014 (0.013, 0.016)	0.016 (0.015, 0.018)	<0.001^3^	<0.001
C14.1	0.012 (0.008, 0.017)	0.012 (0.009, 0.017)	0.519^3^	0.605
C14.2	0.009 (0.006, 0.012)	0.009 (0.006, 0.012)	0.715^3^	0.771
C14OH	0.005 (0.005, 0.005)	0.005 (0.005, 0.006)	0.007^3^	0.019
C16	0.039 (0.034, 0.048)	0.039 (0.033, 0.043)	0.137^3^	0.192
C16.1	0.013 (0.010, 0.015)	0.013 (0.011, 0.015)	0.345^3^	0.439
C16OH	0.033 (0.031, 0.036)	0.038 (0.035, 0.041)	<0.001^3^	<0.001
C16.1OH	0.013 (0.010, 0.016)	0.013 (0.009, 0.017)	0.878^3^	0.899
C18	0.021 (0.018, 0.023)	0.020 (0.017, 0.022)	0.044^3^	0.102
C18.1	0.041 (0.033, 0.054)	0.037 (0.032, 0.046)	0.119^3^	0.178
C18.2	0.019 (0.017, 0.021)	0.018 (0.016, 0.020)	0.068^3^	0.136
C18OH	0.003 (0.002, 0.003)	0.002 (0.002, 0.003)	0.949^3^	0.949
C18.1OH	0.001 (0.001, 0.002)	0.001 (0.001, 0.002)	0.833^3^	0.875

^1^ Mean ± SD; n (%).

^2^ Welch Two Sample t-test.

^3^ Mann-Whitney U test.

#### 3.2.2 Villous tissue

A total of 25 metabolites were identified to be significantly differentially expressed (FDR < 0.05), whereas no statistically significant differences were detected in the expression levels of the other 17 metabolites between the two groups. Most acylcarnitines, such as C16OH, C5DC/C6OH, C10:1, C18, C14OH, C12, C14 and C18OH, were significantly elevated in the missed abortion group, and C10:1 had the highest fold change of 2.448. By contrast, metabolites including C3, C0, C4 and C5 were markedly reduced in the missed abortion group. Regarding amino acid metabolites, ARG and CIT were remarkably increased in the missed abortion group (FDR < 0.05). ORN showed a marginally significant difference (FDR = 0.056) with lower levels compared with the control group. No significant intergroup differences were found in other amino acids, namely ALA, GLY, LEU+ILE+ProOH, MET, PHE, PRO, TYR and VAL (**Table 3**; **Figure 1B**).

**Table 3:** Differential metabolites in villous tissue.

Characteristic	Missed abortion, N = 53^1^	Control group, N = 72^1^	P-value	FDR
ALA	331.507 (246.259, 503.717)	381.235 (232.067, 461.816)	0.974^2^	0.775
ARG	110.471 (77.916, 149.014)	84.770 (66.094, 115.252)	0.006^2^	0.033
CIT	15.156 (10.038, 21.955)	10.976 (6.364, 16.228)	0.008^2^	0.027
GLY	404.255 (266.634, 636.695)	360.204 (252.582, 508.706)	0.247^2^	0.135
LEU+ILE+ProOH	218.315 (171.824, 313.231)	196.495 (143.078, 273.886)	0.205^2^	0.462
MET	42.526 (32.458, 61.904)	41.205 (30.201, 57.918)	0.309^2^	0.562
ORN	30.762 (20.211, 46.656)	45.354 (30.989, 63.585)	0.005^2^	0.056
PHE	83.671 (70.132, 121.249)	79.030 (58.043, 108.779)	0.126^2^	0.362
PRO	135.962 (88.811, 184.246)	122.177 (80.276, 162.888)	0.218^2^	0.233
TYR	78.385 (64.046, 107.887)	69.684 (54.121, 98.659)	0.091^2^	0.234
VAL	153.018 (123.975, 217.098)	147.750 (108.567, 202.592)	0.375^2^	0.54
C0	15.227 (11.130, 19.103)	23.063 (14.905, 33.035)	<0.001^2^	0.008
C2	11.331 (6.998, 18.501)	9.511 (6.479, 13.910)	0.086^2^	0.073
C3	0.524 (0.420, 0.956)	1.189 (0.858, 1.629)	<0.001^2^	0.003
C3DC/C4OH	0.352 (0.216, 0.569)	0.256 (0.184, 0.401)	0.023^2^	0.033
C4	0.582 (0.368, 0.890)	0.908 (0.571, 1.225)	0.002^2^	0.024
C4DC/C5OH	0.101 (0.069, 0.134)	0.122 (0.095, 0.163)	0.002^2^	0.029
C5	0.204 (0.134, 0.383)	0.422 (0.284, 0.638)	<0.001^2^	0.022
C5.1	0.025 (0.016, 0.032)	0.027 (0.021, 0.036)	0.060^2^	0.632
C5DC/C6OH	0.561 (0.419, 0.744)	0.421 (0.373, 0.485)	<0.001^2^	0.001
C6	0.173 (0.138, 0.261)	0.130 (0.110, 0.184)	<0.001^2^	0.024
C6DC	0.175 (0.164, 0.192)	0.167 (0.161, 0.184)	0.078^2^	0.14
C8	0.017 (0.010, 0.031)	0.011 (0.007, 0.016)	<0.001^2^	0.029
C8.1	0.013 (0.009, 0.029)	0.012 (0.010, 0.017)	0.730^2^	0.233
C10	0.009 (0.007, 0.016)	0.007 (0.006, 0.008)	<0.001^2^	0.01
C10.1	0.015 (0.009, 0.020)	0.006 (0.005, 0.009)	<0.001^2^	0.001
C10.2	0.004 (0.004, 0.005)	0.004 (0.003, 0.005)	0.048^2^	0.071
C12	0.005 (0.004, 0.008)	0.003 (0.003, 0.004)	<0.001^2^	0.001
C12.1	0.005 (0.004, 0.006)	0.004 (0.004, 0.005)	<0.001^2^	0.005
C14	0.018 (0.014, 0.021)	0.012 (0.010, 0.015)	<0.001^2^	0.001
C14.1	0.003 (0.003, 0.004)	0.003 (0.002, 0.003)	<0.001^2^	0.054
C14.2	0.003 (0.002, 0.003)	0.003 (0.002, 0.003)	0.035^2^	0.033
C14OH	0.007 (0.005, 0.008)	0.005 (0.004, 0.007)	<0.001^2^	0.001
C16	0.023 (0.014, 0.039)	0.010 (0.008, 0.016)	<0.001^2^	0.025
C16.1	0.005 (0.004, 0.006)	0.003 (0.003, 0.004)	<0.001^2^	0.029
C16OH	0.025 (0.016, 0.034)	0.013 (0.010, 0.019)	<0.001^2^	<0.001
C16.1OH	0.021 (0.014, 0.029)	0.019 (0.013, 0.025)	0.183^2^	0.175
C18	0.021 (0.014, 0.039)	0.010 (0.009, 0.013)	<0.001^2^	0.001
C18.1	0.008 (0.006, 0.013)	0.005 (0.005, 0.007)	<0.001^2^	0.027
C18.2	0.010 (0.007, 0.012)	0.007 (0.005, 0.009)	<0.001^2^	0.001
C18OH	0.003 (0.002, 0.006)	0.001 (0.001, 0.002)	<0.001^2^	0.001
C18.1OH	0.002 (0.001, 0.002)	0.001 (0.001, 0.002)	0.043^2^	0.775

^1^ Mean ± SD; n (%).

^2^ Mann-Whitney U test.

### 3.3 Firth penalized logistic regression

#### 3.3.1 Serum

Due to complete data separation of carnitine metabolites between the two groups, a stable logistic regression model could not be established; therefore, only the intergroup comparison results were presented. For multivariate regression analysis, only amino acid metabolites with model convergence were included for independent correlation analysis. The adjusted odds ratios (ORs) of the 8 amino acid metabolites were all greater than 1, indicating that elevated serum levels of these metabolites were independently associated with an increased risk of missed abortion, acting as independent risk factors (**Table 4**).

**Table 4:** Firth penalized logistic regression analysis of metabolites in serum.

Metabolites	Unadjusted OR (95% CI)	Unadjusted p value	Adjusted OR (95% CI) a	Adjusted p value
ORN	1.138 (1.087~1.197)	<0.001	1.133 (1.083~1.193)	<0.001
TYR	1.243 (1.143~1.366)	<0.001	1.247 (1.146~1.374)	<0.001
LEU+ILE+ProOH	1.067 (1.040~1.100)	<0.001	1.071 (1.043~1.105)	<0.001
ALA	1.028 (1.017~1.042)	<0.001	1.028 (1.016~1.041)	<0.001
VAL	1.047 (1.025~1.072)	<0.001	1.049 (1.026~1.074)	<0.001
PRO	1.055 (1.027~1.087)	<0.001	1.054 (1.025~1.086)	<0.001
CIT	1.408 (1.149~1.757)	0.001	1.418 (1.154~1.773)	0.001
ARG	1.065 (1.019~1.118)	0.004	1.069 (1.022~1.124)	0.003

^a^ Adjusted for age, BMI.

#### 3.3.2 Villous tissue

Univariate analysis demonstrated that C5DC/C6OH, C6, C3DC/C4OH,CIT and ARG were risk factors for missed abortion (OR>1, P<0.05). While C3, C0,C5, C4 and C4DC/C5OH acted as protective factors (OR<1, P<0.05) (**Table 5**).

**Table 5:** Firth penalized logistic regression analysis of metabolites in villous tissue.

Metabolites	Unadjusted OR (95% CI)	Unadjusted p value	Adjusted OR (95% CI) a	Adjusted p value
C5DC/C6OH	121.998 (12.505~1800.691)	<0.001	163.179 (16.048~2536.292)	<0.001
C3	0.310 (0.139~0.614)	<0.001	0.324 (0.146~0.639)	<0.001
C0	0.951 (0.915~0.983)	0.001	0.954 (0.918~0.986)	0.003
C5	0.187 (0.042~0.651)	0.006	0.210 (0.046~0.746)	0.013
C6	241.346 (3.919~25650.940)	0.008	287.860 (4.651~30853.070)	0.006
C4	0.451 (0.211~0.840)	0.010	0.468 (0.219~0.879)	0.016
CIT	1.052 (1.012~1.099)	0.010	1.051 (1.011~1.099)	0.012
C3DC/C4OH	5.029 (1.416~23.217)	0.010	5.261 (1.411~25.499)	0.011
C4DC/C5OH	0.001 (0.000~0.278)	0.012	0.001 (0.000~0.534)	0.023
ARG	1.007 (1.001~1.014)	0.019	1.008 (1.002~1.015)	0.010

^a^ Adjusted for age, BMI.

### 3.4 ROC curves

#### 3.4.1 Serum

**Figure 2:**
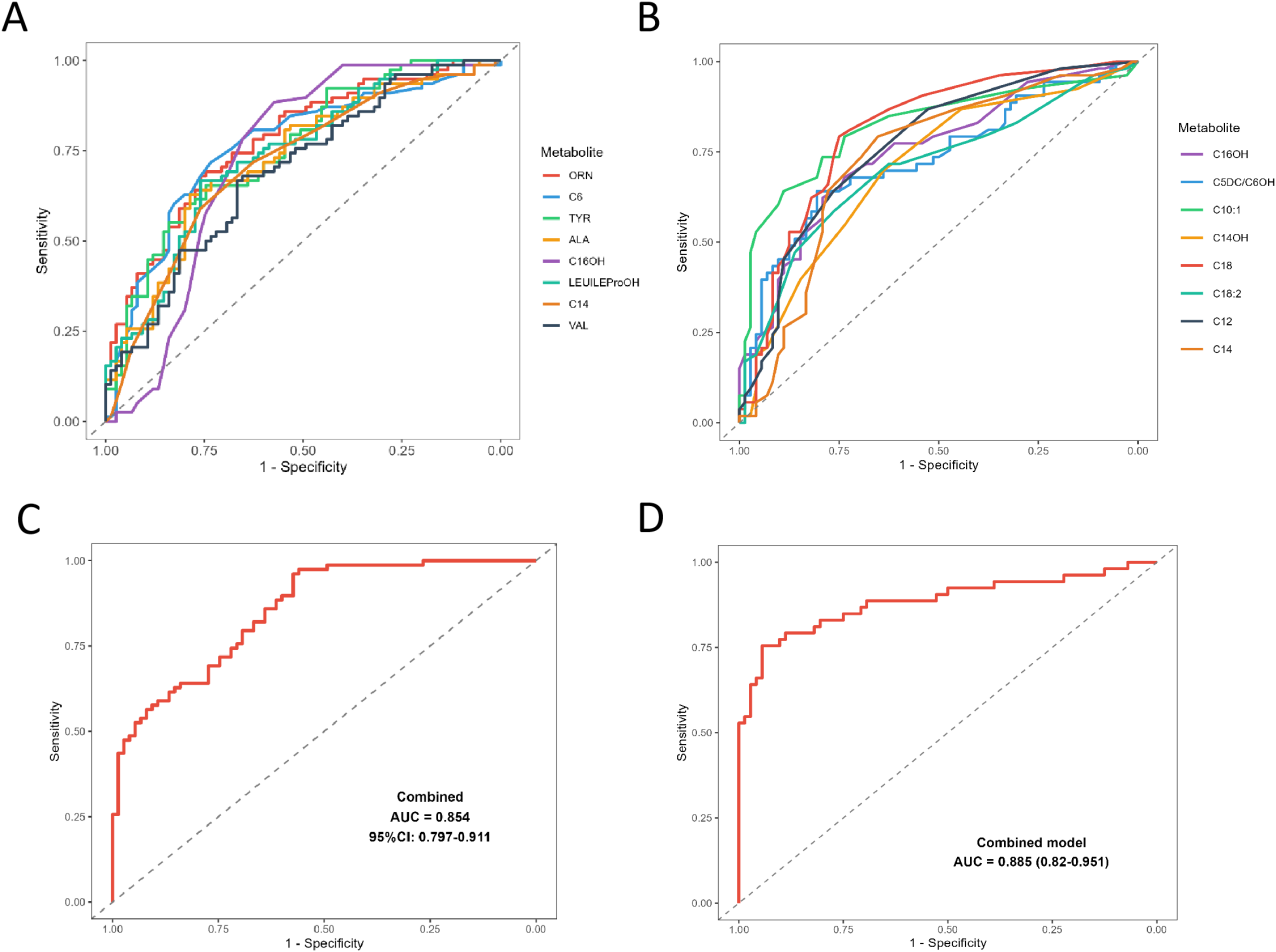
**(A)** ROC curves of the top eight significantly differential metabolites in serumfor the diagnosis of missed abortion. **(B)** ROC curves of the top eight significantly differential metabolites in villous tissue for the diagnosis of missed abortion. **(C)** ROC curve of the combined diagnostic model based on eight differential metabolites in serum. **(D)** ROC curve of the combined diagnostic model based on eight differential metabolites in villous tissue. The area under the curve (AUC) together with 95% confidence intervals were used to evaluate the diagnostic efficacy of each metabolite, and the diagonal line represented the random reference line.

ROC curve analysis revealed that the top 8 metabolites with the most significant differences after FDR correction exhibited satisfactory discriminatory efficiency for missed abortion, with the area under the curve (AUC) ranging from 0.693 to 0.777. Ornithine (ORN) yielded the highest diagnostic efficiency (AUC = 0.777, 95%CI: 0.704-0.849), followed by hexanoylcarnitine (C6, AUC = 0.763, 95%CI: 0.685-0.840) and tyrosine (TYR, AUC = 0.753, 95%CI: 0.677-0.829). These findings indicated that amino acid and carnitine metabolites possess potential diagnostic values, and can serve as auxiliary biomarkers to distinguish missed abortion from normal early pregnancy. Furthermore, a combined diagnostic model was established based on the eight metabolites. The combined model achieved an AUC of 0.854 (95%CI: 0.797-0.911), which was significantly superior to that of any single metabolite. It suggested that this combined model has favorable auxiliary diagnostic performance and could effectively differentiate missed abortion from normal early pregnancy (**Table 6**; **Figures 2A, C**).

**Table 6:** AUCs and 95% CI (Serum).

Metabolites	AUC	95%CI
ORN	0.777	0.704-0.849
C6	0.763	0.685-0.840
TYR	0.753	0.677-0.829
LEU+ILE+ProOH	0.735	0.656-0.814
ALA	0.728	0.647-0.808
C16OH	0.728	0.642-0.814
C14	0.714	0.632-0.795
VAL	0.693	0.610-0.776
Combined Diagnostic Model	0.854	0.797-0.911

#### 3.4.2 Villous tissue

ROC curve analysis was performed on the top eight differentially expressed metabolites ranked by FDR in villous tissues. Each single metabolite exhibited moderate diagnostic efficacy for distinguishing missed abortion, among which C10:1 (AUC = 0.821) and C18 (AUC = 0.808) showed relatively superior performance. The combined diagnostic model established based on these eight metabolites yielded an AUC value of 0.885 (95% CI: 0.820–0.951), which was significantly higher than that of any individual metabolite. These results suggest that the combination of acylcarnitine metabolites in villous tissues can serve as potential auxiliary indicators for differentiating missed abortion (**Table 7**; **Figures 2B, D**).

**Table 7:** AUCs and 95% CI(Villous tissue).

Metabolites	AUC	95%CI
C16OH	0.749	0.661~0.836
C5DC/C6OH	0.729	0.637~0.822
C10:1	0.821	0.740~0.901
C14OH	0.706	0.613~0.798
C18	0.808	0.730~0.885
C18:2	0.706	0.611~0.801
C12	0.767	0.685~0.849
C14	0.732	0.641~0.823
Combined Diagnostic Model	0.885	0.820~0.951

## 4 Discussion

Recent metabolomic studies demonstrated that serum metabolites including amino acids and acylcarnitines are significantly altered in early pregnancy complications such as missed abortion, providing potential biomarkers and insight into metabolic dysregulation mechanisms. For example, Xia et al. used LC-MS to identify differential serum metabolites associated with missed miscarriage (14) and Zhou et al. applied NMR-based metabolomics to characterize distinct metabolic profiles in missed abortion serum (13). Additionally, longitudinal pregnancy metabolomics has shown dynamic changes in amino acids and acylcarnitines during healthy gestation (15), and first-trimester serum acylcarnitines have been linked to other pregnancy metabolic outcomes (16). These findings support the emerging role of altered metabolite pathways in adverse early pregnancy outcomes.

This study conducted targeted metabolomics analysis of serum and villous tissues from women with missed abortion and normal early pregnancy, the findings showed that missed abortion was associated with differences in 17 metabolites in serum samples and 25 metabolites in villous tissue samples from patients in early pregnancy. Firth regression analysis of serum samples revealed that eight amino acids, namely ORN, TYR, LEU+ILE+ProOH, ALA, VAL, PRO, CIT, ARG, were independently associated with an increased risk of missed abortion, acting as independent risk factors. Amino acid metabolism is critical for early embryonic development, supporting trophoblast proliferation, immune tolerance, and energy supply (17). The elevation of circulating amino acids in missed abortion may reflect maternal metabolic dyshomeostasis, potentially impairing early pregnancy maintenance. Villous tissue analysis showed that C5DC/C6OH, C6, C3DC/C4OH, CIT and ARG were risk factors for missed abortion, while C3, C0, C5, C4 and C4DC/C5OH served as protective factors. The carnitine pool plays a vital role in maintaining the body’s energy balance and overall health. Carnitine finely regulates the metabolic pathway through fatty acid transport (18). In this study, the reduction of free short-chain carnitines and the accumulation of medium- and long-chain acylcarnitines in villous tissues from patients with missed abortion indicate impaired β-oxidation, which may lead to energy deficiency, lipotoxicity, and impaired trophoblast viability and differentiation, ultimately resulting in embryonic developmental arrest and missed abortion. Additionally, ARG and CIT were significantly upregulated in villous tissues, consistent with their roles in nitric oxide synthesis and placental angiogenesis (19), their dysregulation may disrupt vascular development and maternal–fetal blood flow.

Notably, only CIT was significantly upregulated in both serum and villous tissues, indicating its potential as a key shared metabolic mediator in missed abortion. CIT is involved in urea cycle and nitric oxide metabolism (20), and its concurrent elevation in serum and villous tissues indicates a coordinated dysregulation of nitrogen metabolism, which may impair maternal metabolic homeostasis and villous angiogenesis. CIT is an α-amino acid generated intracellularly from glutamine metabolism (21); it bypasses hepatic metabolism, enters the systemic circulation, reaches the kidneys, and is converted to arginine, through which it exerts diverse biological effects (22). In the present study, CIT levels were elevated in both the serum and villous tissue of patients with missed abortion. This contrasts with the generally reported protective role of CIT in various diseases, including cardiovascular and metabolic disorders, and suggests that increased CIT may raise the risk of missed abortion. However, the majority of differential metabolites were tissue-specific, with no highly consistent metabolites identified between serum and villous tissues. This discrepancy is biologically reasonable: serum reflects systemic maternal metabolism, while villous tissues directly mirror local embryonic and placental metabolic status. Differences in cellular composition, physiological function, and regulatory pathways between the two matrices inherently lead to distinct metabolic profiles. Furthermore, this study employed a targeted metabolomics approach with limited metabolite coverage, which may also contribute to the lack of overlapping differential metabolites.

ROC curve analysis demonstrated that ORN, C6, and TYR exhibited the highest diagnostic efficacy in serum, while C10:1 and C18 performed best in villous tissues. The combined models of the top eight FDR-ranked metabolites achieved excellent discriminatory performance, with AUC values of 0.886 (serum) and 0.885 (villous tissue), significantly outperforming individual metabolites. These results indicate that both serum amino acid and villous acylcarnitine panels have potential as auxiliary discriminative indicators for missed abortion.

This study has several limitations. First, gestational age was not well-matched between groups, with longer amenorrhea days in the missed abortion group, which may confound metabolic profile comparisons. Second, the sample size was moderate and from a single center, limiting generalizability. Third, targeted metabolomics only detected a predefined set of metabolites, potentially missing key regulatory molecules. Future studies should enroll larger, gestational age-matched cohorts, integrate untargeted metabolomics and multi-omics data, and validate key metabolites in independent cohorts to further elucidate the metabolic mechanisms of missed abortion.

In conclusion, this study confirmed that missed abortion is characterized by serum amino acid hypermetabolism and villous acylcarnitine disorder, reflecting disrupted maternal–fetal metabolic crosstalk. The consistent alteration of CIT in both matrices highlights its potential as a core metabolic indicator. These findings advance our understanding of missed abortion pathogenesis.

## 5 Conclusion

This study performed targeted metabolomics profiling of serum and villous tissues from 78 patients with missed abortion and 75 women with normal early pregnancy undergoing elective termination, to characterize metabolic signatures associated with missed abortion. ROC analysis showed that ORN, C6 and TYR had the highest diagnostic efficacy in serum, while C10:1 and C18 performed best in villous tissue. The combined diagnostic model constructed from the top eight FDR-ranked serum metabolites (ORN, TYR, LEU+ILE+ProOH, ALA, VAL, PRO, CIT, ARG) achieved an AUC of 0.886, significantly outperforming any single metabolite, indicating good auxiliary diagnostic value for missed abortion. The combined model based on the top eight FDR-ranked villous tissue metabolites (C16OH, C5DC/C6OH, C10:1, C14OH, C18, C18:2, C12, C14) yielded an AUC of 0.885, with discriminatory ability superior to individual metabolites, suggesting that the villous acylcarnitine panel may serve as a potential auxiliary discriminative indicator for missed abortion. Notably, only CIT was consistently and significantly upregulated in both serum and villous tissue, while other differential metabolites were tissue-specific. The lack of highly consistent metabolites between the two sample types may be related to the limited coverage of the targeted metabolomics assay. These findings provide new insights into the metabolic pathogenesis of missed abortion.

## Data Availability

The original contributions presented in the study are included in the article/supplementary material. Further inquiries can be directed to the corresponding author.

## References

[1] Fang Q Sang L Du S Wang R Wu H Yang L . Vitamin D, homocysteine, and thyroid dysfunction as risk factors for missed abortion: A retrospective risk factor analysis. Ijwh. (2025) 17:1587–96. doi: 10.2147/IJWH.S507470 40470006 PMC12135947

[2] Liu S Song L Cram DS Xiong L Wang K Wu R . Traditional karyotyping vs copy number variation sequencing for detection of chromosomal abnormalities associated with spontaneous miscarriage. Ultrasound Obstet Gynecol. (2015) 46:472–7. doi: 10.1002/uog.14849 25767059

[3] Gong G Yin C Huang Y Yang Y Hu Z Zhu Z . A survey of influencing factors of missed abortion during the two-child peak period. J Obstet Gynaecol. (2021) 41:977–980. doi: 10.1080/01443615.2020.1821616 33241701

[4] Oliveira RV Simionato AVC Cass QB . Enantioselectivity effects in clinical metabolomics and lipidomics. Molecules. (2021) 26:5231. doi: 10.3390/molecules26175231 34500665 PMC8433918

[5] Diaz SO Pinto J Graça G Duarte IF Barros AS Galhano E . Metabolic biomarkers of prenatal disorders: An exploratory NMR metabonomics study of second trimester maternal urine and blood plasma. J Proteome Res. (2011) 10:3732–42. doi: 10.1021/pr200352m 21649438

[6] Bahado-Singh RO Akolekar R Mandal R Dong E Xia J Kruger M . Metabolomics and first-trimester prediction of early-onset preeclampsia. J Maternal-Fetal Neonatal Med. (2012) 25:1840–7. doi: 10.3109/14767058.2012.680254 22494326

[7] Wallace M Cottell E Gibney MJ McAuliffe FM Wingfield M Brennan L . An investigation into the relationship between the metabolic profile of follicular fluid, oocyte developmental potential, and implantation outcome. Fertility Sterility. (2012) 97:1078–1084.e8. doi: 10.1016/j.fertnstert.2012.01.122 22365382

[8] Liu X Wang X Sun H Guo Z Liu X Yuan T . Urinary metabolic variation analysis during pregnancy and application in gestational diabetes mellitus and spontaneous abortion biomarker discovery. Sci Rep. (2019) 9:2605. doi: 10.1038/s41598-019-39259-2 30796299 PMC6384939

[9] Youssef L Crovetto F Simoes RV Miranda J Paules C Blasco M . The interplay between pathophysiological pathways in early-onset severe preeclampsia unveiled by metabolomics. Life. (2022) 12:86. doi: 10.3390/life12010086 35054479 PMC8780941

[10] Huang Y Tu M Qian Y Ma J Chen L Liu Y . Age-dependent metabolomic profile of the follicular fluids from women undergoing assisted reproductive technology treatment. Front Endocrinol. (2022) 13:818888. doi: 10.3389/fendo.2022.818888 35250874 PMC8888916

[11] Wang X Zhao X Zhao J Yang T Zhang F Liu L . Serum metabolite signatures of epithelial ovarian cancer based on targeted metabolomics. Clin Chim Acta. (2021) 518:59–69. doi: 10.1016/j.cca.2021.03.012 33746017

[12] Fei H Hou J Wu Z Zhang L Zhao H Dong X . Plasma metabolomic profile and potential biomarkers for missed abortion. BioMed Chromatogr. (2016) 30:1942–52. doi: 10.1002/bmc.3770 27229294

[13] Wu Z Jin L Zheng W Zhang C Zhang L Chen Y . NMR-based serum metabolomics study reveals a innovative diagnostic model for missed abortion. Biochem Biophys Res Commun. (2018) 496:679–85. doi: 10.1016/j.bbrc.2018.01.096 29353036

[14] Xia L Zhao H Shan L Ma X An P Duan X . Using liquid chromatography and mass spectrometry to predict potential biomarkers for missed miscarriage and its metabolic pathways in a tertiary center: A cross‐sectional analytic study. Intl J Gynecology Obste. (2024) 166:312–25. doi: 10.1002/ijgo.15417 38445380

[15] Mitro SD Wu J Rahman ML Cao Y Zhu Y Chen Z . Longitudinal plasma metabolomics profile in pregnancy-a study in an ethnically diverse U.S. pregnancy cohort. Nutrients. (2021) 13:3080. doi: 10.3390/nu13093080 34578958 PMC8471130

[16] Nevalainen J Sairanen M Appelblom H Gissler M Timonen S Ryynänen M . First-trimester maternal serum amino acids and acylcarnitines are significant predictors of gestational diabetes. Rev Diabetes Stud. (2016) 13:236–45. doi: 10.1900/RDS.2016.13.236 28278310 PMC5734224

[17] Lin CJ Geng GX Peng ZR Huang XT Wu LL Xu, YQ . Characteristics of amino acid metabolism in preterm infants in Guangxi, China. Zhongguo Dang Dai Er Ke Za Zhi. (2022) 24:162–168. doi: 10.7499/j.issn.1008-8830.2109149 35209981 PMC8884053

[18] Xiang F Zhang Z Xie J Xiong S Yang C Liao D . Comprehensive review of the expanding roles of the carnitine pool in metabolic physiology: Beyond fatty acid oxidation. J Transl Med. (2025) 23:324. doi: 10.1186/s12967-025-06341-5 40087749 PMC11907856

[19] Jahoor F Badaloo A Villalpando S Reid M Forrester T . Arginine flux and intravascular nitric oxide synthesis in severe childhood undernutrition. Am J Clin Nutr. (2007) 86:1024–1031. doi: 10.1093/ajcn/86.4.1024 17921380

[20] Imbard A Bouchereau J Arnoux JB Brassier A Schiff M Bérat CM . Citrulline in the management of patients with urea cycle disorders. Orphanet J Rare Dis. (2023) 18:207. doi: 10.1186/s13023-023-02800-8 37480106 PMC10362745

[21] Curis E Nicolis I Moinard C Osowska S Zerrouk N Bénazeth S . Almost all about citrulline in mammals. Amino Acids. (2005) 29:177. doi: 10.1007/s00726-005-0235-4 16082501

[22] Wu G Morris SM . Arginine metabolism: Nitric oxide and beyond. Biochem J. (1998) 336:1–17. doi: 10.1042/bj3360001 9806879 PMC1219836

