# Supplementary material for: Analysis of metabolites in serum and villous tissue of missed abortion patients based on targeted metabolomics
Source: Front Endocrinol (Lausanne). 2026 Jun 18;17:1782638. doi: 10.3389/fendo.2026.1782638 (PMC13322866; doi:10.3389/fendo.2026.1782638)
Supplement: Supplementary file 1 [file Table1.docx]

Supplementary Material

# Supplementary Table

# Supplementary Table 1 Metabolite Abbreviation Reference

| **Abbreviation** | **Full Name** |
| --- | --- |
| ALA | Alanine |
| ARG | Arginine |
| CIT | Citrulline |
| GLY | Glycine |
| LEU+ILE+ProOH | Leucine + Isoleucine + Proline |
| MET | Methionine |
| ORN | Ornithine |
| PHE | Phenylalanine |
| PRO | Proline |
| TYR | Tyrosine |
| VAL | Valine |
| C0 | Free Carnitine |
| C2 | Acetylcarnitine |
| C3 | Propionylcarnitine |
| C3DC/C4OH | Malonylcarnitine |
| C4 | Butyrylcarnitine |
| C4DC/C5OH | Methylmalonylcarnitine |
| C5 | Isovalerylcarnitine |
| C5:1 | Tiglylcarnitine |
| C5DC/C6OH | Glutarylcarnitine |
| C6 | Hexanoylcarnitine |
| C6-DC | Adipylcarnitine |
| C8 | Octanoylcarnitine |
| C8:1 | Octenoylcarnitine |
| C10 | Decanoylcarnitine |
| C10:1 | Decenoylcarnitine |
| C10:2 | Decadienoylcarnitine |
| C12 | Lauroylcarnitine |
| C12:1 | Lauroleoylcarnitine |
| C14 | Myristoylcarnitine |
| C14:1 | Myristoleoylcarnitine |
| C14:2 | Myristolenoylcarnitine |
| C14OH | 3-Hydroxymyristoylcarnitine |
| C16 | **Palmitoylcarnitine** |
| C16:1 | **Palmitoleoylcarnitine** |
| C16:1OH | **3-Hydroxypalmitoleoylcarnitine** |
| C16OH | **3-Hydroxypalmitoylcarnitine** |
| C18 | **Stearoylcarnitine** |
| C18:1 | **Oleoylcarnitine** |
| C18:2 | **Linoleoylcarnitine** |
| C18:1-OH | **3-Hydroxyoleoylcarnitine** |
| C18-OH | **3-Hydroxystearoylcarnitine** |
